# Efficacy of a compulsory homework programme for increasing physical activity and healthy eating in children: the healthy homework pilot study

**DOI:** 10.1186/1479-5868-8-127

**Published:** 2011-11-15

**Authors:** Scott Duncan, Julia C McPhee, Philip J Schluter, Caryn Zinn, Richard Smith, Grant Schofield

**Affiliations:** 1 Centre for Physical Activity and Nutrition, AUT University, New Zealand; 2 Department of Public Health and General Practice, University of Otago, Christchurch, New Zealand; 3 School of Public Health and Psychosocial Studies, AUT University, New Zealand; 4 School of Nursing and Midwifery, University of Queensland, Australia; 5 National Institute of Education, Singapore

## Abstract

**Background:**

Most physical activity and nutrition interventions in children focus on the school setting; however, evidence suggests that children are less active and have greater access to unhealthy food at home. The aim of this pilot study was to examine the efficacy of a compulsory homework programme for increasing physical activity and healthy eating in children.

**Methods:**

The six-week 'Healthy Homework' programme and complementary teaching resource was developed under the guidance of an intersectoral steering group. Eight senior classes (year levels 5-6) from two diverse Auckland primary schools were randomly assigned into intervention and control groups. A total of 97 children (57 intervention, 40 control) aged 9-11 years participated in the evaluation of the intervention. Daily step counts were monitored immediately before and after the intervention using sealed multiday memory pedometers. Screen time, sports participation, active transport to and from school, and the consumption of fruits, vegetables, unhealthy foods and drinks were recorded concurrently in a 4-day food and activity diary.

**Results:**

Healthy Homework resulted in a significant intervention effect of 2,830 steps.day^-1 ^(95% CI: 560, 5,300, P = 0.013). This effect was consistent between sexes, schools, and day types (weekdays and weekend days). In addition, significant intervention effects were observed for vegetable consumption (0.83 servings.day^-1^, 95% CI: 0.24, 1.43, P = 0.007) and unhealthy food consumption (-0.56 servings.day^-1^, 95% CI: -1.05, -0.07, P = 0.027) on weekends but not weekdays, with no interactions with sex or school. Effects for all other variables were not statistically significant regardless of day type.

**Conclusions:**

Compulsory health-related homework appears to be an effective approach for increasing physical activity and improving vegetable and unhealthy food consumption in children. Further research in a larger study is required to confirm these initial results.

## Background

Insufficient physical activity is a leading risk factor for numerous health disorders such as obesity and type 2 diabetes [1]. While methodological differences make it difficult to compare secular trends in children's free-living physical activity, there is evidence that organised physical activity is declining in many countries [2]. Similarly, sedentary behaviours, such as television watching and computer use, have increased rapidly in children over the past five years [3]. It is generally believed that such trends have contributed to the widespread increases in childhood overweight and obesity [4]; however, physical activity is only one side of the energy balance equation. Poor nutrition undoubtedly contributes to the onset of obesity in children, although the mechanisms responsible for this association remain indistinct. Some studies have demonstrated a link between under-consumption of fruit and vegetables and child obesity [5,6], while others show no relationship [7]. Likewise, the over-consumption of energy-dense foods and drinks has been associated with child obesity in some [8-10] but not all studies [11-13]. Regardless of the specific pathways to chronic disease, physical activity and dietary patterns tend to track across the lifespan [14-19]; therefore, it makes sense to correct unhealthy habits before they have a lasting impact. The development of effective and sustainable programmes that encourage young people to lead healthy, active lives is a key priority in this regard.

The majority of lifestyle interventions for children have focused on the promotion of healthy behaviours while children are at school; however, there is a growing body of evidence suggesting that children are more likely to be inactive and consume unhealthy foods when at home. Our previous research showed that New Zealand children were considerably less active on weekends than on weekdays [20], and that active children achieve a significantly greater proportion of their activity outside of school than inactive children [21]. This is consistent with international data that support the promotion of physical activity in the home environment [22-24]. Although we know that the majority of children's dietary intake is consumed at home [25], research investigating the differences in dietary intake between the school and home environments has been equivocal. A recent study reported that New Zealand children are more likely to consume high cholesterol foods and soft drinks on non-school days than on school days [26]. Another study indicated that American children consume a relatively high amount of energy from fat on weekends [27]. Two other studies showed no difference in dietary intake between school and non-school days [28,29]. In any case, reviews of interventions to promote physical activity [30,31] and correlates of dietary behaviour [32] in children and adolescents concluded that programmes that involve families are more likely to be effective than those that do not. Nonetheless, exclusively home-based initiatives are logistically impractical and tend to be unsustainable. A more feasible approach is to use schools to access children for the purpose of encouraging healthy behaviour in the home environment.

Several studies have endeavoured to promote physical activity and/or healthy eating outside of school by incorporating homework components into school-based interventions. Results have been mixed, with some showing positive effects [33-40] and others showing no effect [41-46]. However, it is difficult to determine the contributions of homework given that all studies employed multiple approaches, and many were limited by low statistical power [33], self- or proxy-reporting of physical activity [33-35,41,43,45], or relatively minor homework components [33,34,36,37,39,40,44,45]. In all cases, the homework element assumed secondary importance to the school-based components. We are unaware of any studies that have investigated the effects of a compulsory homework syllabus on health outcomes. The aim of this pilot study was to examine the efficacy of a compulsory homework programme for increasing physical activity and healthy eating in children.

## Methods

### Intervention

Healthy Homework was developed between August and December 2008 under the guidance of an advisory committee comprised of experienced health and education professionals. The primary aim of the intervention was to improve physical activity and dietary behaviours in participating children. Ten key sub-behaviours were identified for intervention: (1) walking frequency, (2) television usage, (3) participation in sport, (4) participation in informal games, (5) participation in fitness activities, (6) fruit and vegetable consumption, (7) breakfast consumption, (8) fluid intake, (9) food labelling knowledge, and (10) food preparation knowledge. Table 1 describes the behaviour change techniques used to modify the key determinants (adapted from Abraham and Michie [47]).

**Table 1:** Description of behaviour change techniques and strategies implemented in the Healthy Homework Pilot Study

Behaviour Change Technique	Theoretical Basis	Key behavioural determinant	Intervention strategy
Provide information about behaviour-health link	IMB	Awareness (personal)	Children were provided with information about the positive health outcomes associated with various healthy behaviours throughout the programme.
Provide information on consequences	TRA, TPB, ScogT, IMB	Awareness (personal)	The beneficial consequences of specific physical activity and healthy eating patterns were reinforced throughout the programme.
Prompt intention formation	TRA, TPB, ScogT, IMB	Attitudes (personal)	At the completion of the programme, children were encouraged to make long-term behavioural resolutions related to the tasks they had accomplished during the programme.
Prompt barrier identification	SCogT	Knowledge (personal)	Several topics required the children to identify common barriers to healthy behaviours and how those barriers might be overcome in the context of their lives.
Set graded tasks	SCogT	Knowledge (personal)	Children were required to complete at least one out of three homework tasks that promoted increased knowledge of a given topic. When one task was completed, children were encouraged to complete all of the remaining tasks to supplement their knowledge.
Provide instruction	SCogT	Availability of information (environmental)	Detailed instruction for each topic was provided in-class by the teacher and via the homework booklets.
Prompt specific goal setting	CT	Self-efficacy (personal)	Each homework task had a specific goal that children could achieve. Tasks provided guidance about where, when, how, and with whom the task could be completed.
Prompt review of behavioural goals	CT	Teacher regulation (environmental)	Each week the teacher was required to review each child's homework tasks from the previous week (individually) and discuss any facilitators or barriers to completion (as a group). Children were given advice about how to complete any unfinished tasks.
Provide feedback on performance	CT	Awareness (personal)	At the completion of the programme, children and their parents received a feedback form that detailed all changes to physical activity and dietary behaviour taken during the evaluation.
Provide contingent rewards	OC	Self-efficacy (personal)	Children received rubber wristbands if they completed their homework requirements for a given week (at least one physical activity and one nutrition task). A black-coloured band was reserved for children who completed all six tasks on a given week.
Prompt practice	OC	Skills (personal)	The majority of homework tasks required multiple sessions or practice of a behaviour.
Provide opportunities for social comparison	SCompT	Subjective norms (social)	Many in-class activities were based on practical group tasks that required children to observe and compare their behaviour against others. Children were encouraged to support each other to complete tasks.
Plan social support or social change	Social support theories	Family support (social)	A large number of homework tasks recommended participation as a family. Messages for the parents reinforcing the benefits of family support were embedded in homework tasks.

IMB = information-motivation-behavioural skills model; TRA = theory of reasoned action; TPB = theory of planned behaviour; SCogT = social-cognitive theory; CT = control theory; OC = operant conditioning.

The final programme consisted of a six-week homework schedule complemented by an in-class teaching resource, and was designed to support the achievement objectives associated with Level 3 of the New Zealand Health and Physical Education Curriculum [48]. Each child received a homework booklet organised into five physical activity and five nutrition topics: Week 1, walking and fruit/vegetables; Week 2, television and breakfast; Week 3, sports and drinks; Week 4, fun games and food shopping; Week 5, fitness and cooking. Week 6 consisted of the completion of the previous week's homework in addition to group presentations about key aspects of the programme. Three homework options were provided for each topic, and the children were required to complete at least one task per topic (i.e., at least two tasks per week). Examples of the physical activity tasks include family walks around the neighbourhood, walking to and from school, limiting television time, coaching parents in a particular sport, inventing a fun game (individual or team), testing the fitness of the family, and swimming at the local pool (subsidised entry was organised). Examples of the nutrition tasks include eating at least five servings of fruit or vegetables, preparing and eating a healthy breakfast, using a water bottle throughout the day, reducing consumption of unhealthy foods and drinks, comparing food labels when shopping, helping to prepare a healthy dinner, and preparing a healthy lunch box. Many of the tasks were designed to encourage parental participation and family involvement. Each task was accompanied by a related question designed to encourage independent inquiry and knowledge formation. Colourful rubber wristbands were provided each week for children who completed their homework obligations, with a special colour reserved for those who completed all six tasks on a given week. Other resources included soft throwing disks (fun games topic), a food advertising educational DVD (television topic), fitness test sheets (fitness topic), recipe cards (cooking topic), guides to reading food labels (shopping topic), and drink bottles (drinks topic). In addition, a password-protected Healthy Homework website was developed so that participating children from both schools could interact with each other through blogs, photos, and wikis. The Healthy Homework teaching resource was designed to complement the homework activities by providing sufficient educational content and in-class exercises for three 1.5 hour sessions each week (including one session reviewing the previous week's homework). Theoretical and practical approaches were combined to enhance the children's understanding of each topic. Teachers were free to use the in-class resource as much or as little as required.

### Participants

Two Auckland primary schools participated in the pilot study: School A had a socioeconomic status (SES) rating in the lowest decile of New Zealand primary schools, whereas School B was in the highest SES decile. Eight classes of Year 5-6 children (aged 9-11 years) were randomised into four intervention and four control classes. All children in the intervention classes completed the Healthy Homework module as part of school policy; however, parental consent was required before children were able to participate in the evaluation of the intervention. Consent was obtained for 100 of the 216 children initially selected (46.3%). Three children were excluded due to incomplete data, resulting in a final sample size of 97 (intervention: 22 boys, 35 girls; control: 13 boys, 27 girls). The ethnic composition of the sample was 48.5% European, 32.0% Pacific Island, 8.2% Māori, 6.2% Asian, and 5.2% from other ethnicities. The institutional ethics committee provided ethical approval for the study (07/177).

### Instruments and Procedures

Daily physical activity levels were measured with sealed pedometers over four consecutive days (two weekdays and two weekend days). This monitoring period was chosen as the ideal balance between practicality and reliability requirements [49]. Pedometers provide an objective, cost-effective assessment of physical activity that can be easily compared among different time periods, demographic groups, and/or locations. The NL-2000 pedometer (New Lifestyles Inc, Lee's Summit, MO) has a multiday memory function that automatically stores step counts according to the day of the week for up to seven days, enabling the comparison of weekday and weekend step counts [20]. Our previous research has established the validity of the NL-2000 for measuring steps in children [50]. Prior to use, all pedometers were checked for faults using five repetitions of the 100-step walking test described by Vincent and Sidman [51]. Instrumental error did not exceed 3% in any of the pedometers. Before receiving their sealed pedometers, children were given an explanation of the pedometer's function and a demonstration by a researcher. Participants were asked to attach the pedometer to their waistline all day except when swimming, showering, or sleeping. To assess participant compliance outside of the school environment, children maintained a 4-day diary in which they were asked to note how many hours they did not wear the pedometer each day. Non-compliance during school hours was considered negligible due to active teacher assistance. Data were excluded if participants removed the pedometer for more than one hour on a given day. Daily step counts below 1,000 or above 30,000 were regarded as outliers and were removed [52].

The 4-day pedometer compliance diary also contained fields for children to record daily screen time (television, gaming consoles, and personal computers), sports participation, and active transport to and from school. In addition, a food diary was issued for children to record the type and quantity of all foods and drinks consumed over the four-day period. A food diary is a daily record of all the food and fluid consumed over a specified time; a blank template for the required days is provided. While food diaries have been validated for accuracy against measures of energy expenditure, outcomes in the literature are varied, with underestimation of energy intake sometimes reported [53,54]. Despite this, we chose to use food diaries so that we could compare both the quality and quantity of food and fluid intake on weekdays and weekend days. Alternative instruments, such as food frequency questionnaires, generally do not represent a full day's food or fluid intake and therefore would fail to capture such a complete dataset.

All participants were given a detailed explanation about how to correctly fill in the diaries, and parents were given written instructions to assist their child in completing the diaries accurately and to a sufficient level of detail. Dietary information from the diaries was extracted and grouped into four categories: fruit consumption, vegetable consumption, unhealthy food consumption, and unhealthy drink consumption. Food and drinks were defined as unhealthy in accordance with the 'occasional foods' tier of the three-tiered New Zealand Food and Beverage classification system. Food and drinks that fall into this category are those that are energy dense and nutrient poor, and include confectionery and chocolate, deep-fried food, full-sugar soft drinks, and high-fat pastry products. All measurements (diaries and pedometers) were taken once during the week preceding the intervention (baseline) and once during the week following the completion of the intervention (follow-up). Both schools were assessed over the same time period (May-June 2009).

### Statistical Analyses

Medians and interquartile ranges for all variables were generated with weekday and weekend data presented separately along with an overall weighted mean (five weekdays to two weekend days). Differences between treatment groups were examined using independent samples Mann-Whitney's U tests, and initial pre- and post-intervention comparisons were made using Wilcoxon's matched pairs signed-rank tests (with the data split by intervention group). A normal generalized estimating equation (GEE) model on square-root transformed step counts was used to detect an intervention effect after accounting for day type, school year, school, class, sex, and ethnicity. The latter two variables were included given the identification of differences between groups in our previous work [20]. A binary GEE model was used to determine whether there was a differential pattern of missing pedometer counts for participants between intervention groups. Treatment group effects for all other variables (screen time, sports participation, active transport, fruit consumption, vegetable consumption, unhealthy food consumption, and unhealthy drink consumption) were examined using analysis of covariance adjusted for regression to the mean. All analyses were performed using Stata version 11.0 (StataCorp, College Station, TX, USA), and α = 0.05 defined statistical significance for all tests.

## Results

### Preliminary analyses

Table 2 shows the physical activity and dietary patterns of the intervention and control groups at baseline and follow-up. The only significant differences between intervention and control groups were for weekend steps at follow-up (P = 0.016) and unhealthy drink consumption at baseline (P = 0.037). In the intervention group, significant increases between baseline and follow-up were observed for weekday steps (P = 0.001), overall steps (P = 0.020), and weekend vegetable consumption (P < 0.001), whereas decreases were observed for unhealthy food consumption on weekdays (P = 0.017), weekends (P < 0.001), and overall (P = 0.001), and for unhealthy drink consumption on weekdays (P = 0.006) and overall (P = 0.010). In the control group, significant pre-post increases were observed for weekday sports participation (P = 0.010), with vegetable consumption and active transport time decreasing on weekdays (P = 0.035 and P = 0.028, respectively).

**Table 2:** Median and interquartile range of the physical activity and dietary variables for the intervention and control groups.

	Intervention			Control		
	*N*	Baseline	Follow-up	*N*	Baseline	Follow-up
Physical activity (steps.day^-1^)						
Weekday	47	10,700 (8,420, 12,150)	12,290 (9,990, 16,270)^‡^	35	11,460 (9,110, 13,660)	10,100 (7,670, 14,160)
Weekend	38	7,940 (5,760, 11,770)	8,670 (6,460, 11,960)	22	8,160 (6,410, 11,470)	7,090 (4,690, 9,220)*
Overall	35	10,790 (8,200, 12,160)	11,790 (9,670, 15,680)^†^	21	11,240 (9,420, 12,860)	9,910 (7,500, 12,700)
Screen time (h.day^-1^)						
Weekday	30	1.00 (0.21, 1.75)	0.83 (0.17, 1.50)	21	0.71 (0.50, 1.68)	1.00 (0.31, 2.38)
Weekend	30	1.29 (0.32, 2.56)	1.75 (0.86, 2.45)	21	1.61 (0.88, 3.19)	1.75 (0.75, 3.50)
Overall	30	1.25 (0.36, 1.93)	1.05 (0.63, 1.52)	21	1.09 (0.59, 2.04)	1.25 (0.39, 2.64)
Sports participation (h.day^-1^)						
Weekday	27	0.50 (0.09, 0.75)	0.75 (0.44, 1.21)	22	0.48 (0.09, 0.76)	0.82 (0.31, 1.00)^†^
Weekend	26	0.67 (0.13, 1.44)	0.58 (0.01, 1.81)	21	0.58 (0.04, 1.33)	0.75 (0.17, 1.42)
Overall	26	0.64 (0.14, 1.01)	0.71 (0.46, 1.28)	21	0.52 (0.14, 0.90)	0.81 (0.41, 1.14)
Active transport to/from school (h.day^-1^)						
Weekday	19	0.17 (0.00, 0.33)	0.13 (0.00, 0.44)	13	0.15 (0.00, 0.46)	0.00 (0.00, 0.29)^†^
Fruit consumption (servings.day^-1^)						
Weekday	33	1.25 (0.94, 1.55)	1.50 (1.00, 2.25)	26	1.00 (0.38, 1.81)	1.50 (0.69, 2.00)
Weekend	33	0.50 (0.00, 1.50)	1.00 (0.50, 1.50)	26	0.50 (0.19, 1.50)	0.50 (0.19, 1.31)
Overall	33	1.07 (0.70, 1.75)	1.43 (0.86, 2.11)	26	1.06 (0.58, 1.43)	1.14 (0.69, 1.66)
Vegetable consumption (servings.day^-1^)						
Weekday	33	1.00 (0.50, 1.88)	1.00 (0.50, 2.00)	26	1.00 (0.50, 2.00)	0.93 (0.00, 1.50)^†^
Weekend	33	0.50 (0.00, 1.00)	2.00 (0.75, 2.50)^‡^	26	1.00 (0.38, 2.25)	1.25 (0.38, 1.81)
Overall	33	0.79 (0.36, 1.68)	1.43 (0.83, 1.93)	26	1.13 (0.47, 1.81)	0.93 (0.36, 1.57)
Unhealthy food consumption (servings.day^-1^)						
Weekday	33	2.00 (1.50, 3.00)	1.50 (0.50, 2.75)^†^	26	2.25 (1.00, 3.50)	1.50 (1.00, 3.13)
Weekend	33	2.00 (1.00, 3.50)	1.00 (0.50, 1.50)^‡^	26	2.00 (1.00, 2.50)	1.50 (1.00, 2.50)
Overall	33	2.07 (1.43, 3.00)	1.21 (0.68, 2.43)^‡^	26	1.90 (1.27, 3.43)	1.68 (1.24, 2.63)
Unhealthy drink consumption (servings.day^-1^)						
Weekday	33	1.00 (0.00, 1.50)	0.00 (0.00, 0.50)^‡^	26	0.00 (0.00, 1.00)*	0.00 (0.00, 0.50)
Weekend	33	1.00 (0.00, 2.00)	0.50 (0.00, 1.25)	26	0.50 (0.00, 1.00)	0.00 (0.00, 1.00)
Overall	33	0.93 (0.07, 1.79)	0.29 (0.00, 0.79)^†^	26	0.29 (0.00, 0.88)	0.29 (0.00, 0.79)

*Significantly different from intervention (P < 0.05). ^†^Significantly different from baseline (P < 0.05); ^‡^Significantly different from baseline (P < 0.01).

### Pedometer analyses

**Figure 1:**
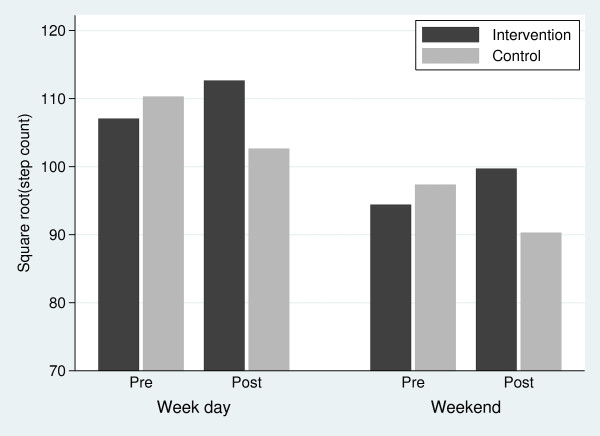
**Summary of the adjusted multivariable model coefficients for square root step counts**.

The distribution of raw step counts departed from normality (Shapiro-Wilk's test; P < 0.001), which was corrected with square-root transformed step count data (Shapiro-Wilk's test; P = 0.261). GEE model analyses of transformed step count data showed no significant effects of school (P = 0.328), school year (P = 0.970), class (P = 0.973), or ethnicity (P = 0.541), and these variables were not considered further. The final adjusted multivariable model included group (intervention/control), period (pre/post), sex (male/female), and day type (weekday/weekend) variables (Figure 1). While the analyses were performed on square-root step counts, the estimates presented below were first transformed back into raw step counts for easier interpretation. Results showed that the mean step count in the control group non-significantly declined from 10,990 (95% CI: 9,860, 12,190) pre-intervention to 9,510 (7,860, 11,330) post-intervention (P = 0.062), whereas the mean step count in the intervention group non-significantly increased from 10,350 (9,340, 11,410) pre-intervention to 11,480 (10,090, 12,960) post-intervention (P = 0.113). Overall, these differences corresponded to a significant intervention effect of 2,830 (560, 5,300) steps.day^-1 ^(P = 0.013). Our results also indicated that (1) there was an independent sex effect with boys averaging 2,500 (95% CI: 740, 4,390) more steps.day^-1 ^than girls over the duration of the study (P = 0.005), and (2) there was an independent day effect with 2,530 (1,580, 3,420) fewer steps on weekend days compared with weekdays over the duration of the study (P < 0.001). However, there were no significant interactions between day × sex (P = 0.310), group × sex (P = 0.851), period × sex (P = 0.150), period × day (P = 0.102), group × period × day (P = 0.763), or group × period × sex (P = 0.811). The Shapiro-Wilk test showed that the residuals did not depart from normality (P = 0.877).

Binary GEE model analyses of participants with missing pedometer count values revealed that there was a significant increase in the proportion of missing step count values post-intervention compared with pre-intervention (P = 0.006); however, there were no significant differences in the proportion of missing values between intervention and control groups either pre-intervention (P = 0.905) or post-intervention (P = 0.481). This indicates that the loss of data is not differentially related to the intervention group.

### Activity and food diary analyses

**Figure 2:**
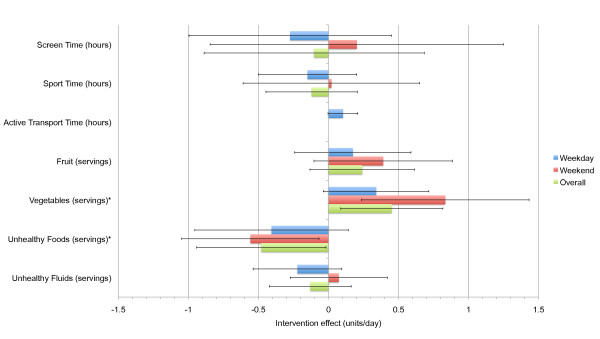
**Intervention effects (± 95% CI) for selected physical activity and dietary behaviours**. *Significant intervention effect for weekends and overall (P < 0.05).

Figure 2 shows the intervention effects derived from multiple analysis of covariance procedures after adjustment for regression to the mean. Positive effects were detected for vegetable consumption on weekends (0.83 servings.day^-1^, 95% CI: 0.24, 1.43, P = 0.007) and overall (0.45 servings.day^-1^; P = 0.016; 95% CI: 0.09, 0.82), with no significant effect on weekdays. In addition, negative effects were detected for unhealthy food consumption on the weekends (-0.56 servings.day^-1^, 95% CI: -1.05, -0.07, P = 0.027) and overall (-0.48 servings.day^-1^; P = 0.042; 95% CI: -0.94, -0.02), but not on weekdays. No significant interactions with sex or school were observed for either vegetable or unhealthy food consumption. Further, no significant intervention effects or interactions were observed for screen time, active transportation to and from school, sports participation, fruit consumption, and unhealthy fluid consumption on weekdays, weekends, or overall.

## Discussion

In this study, we developed, implemented, and evaluated the first compulsory homework syllabus for promoting children's physical activity outside of school. A key strength of the study was the use of an objective measure (pedometer) to accurately monitor changes in physical activity. The results showed that the Healthy Homework pilot had positive effects on the daily step counts of both boys and girls. The intervention effect of 2,830 steps.day^-1 ^corresponds to over 25% more activity each day (based on the sample mean pre-intervention). This effect was driven by both an increase of 1,100 steps.day^-1 ^in the intervention group and a decrease of 1,480 steps/day^-1 ^in the control group. While the reasons for the decrease in the control group are unknown, it appears the programme had a protective effect that precluded a similar decrease in the intervention group. Furthermore, the proportion of children achieving step count targets directly related to the prevention of excess body fat (16,000 steps.day^-1 ^for boys, 13,000 steps.day^-1 ^for girls [55]) increased from 8.6% to 31.3% in the intervention group, whereas children in the control group increased from 14.3% to 16.7%. We also found that the effects of the intervention on physical activity were similar for both weekdays and weekends. This is a noteworthy finding given that children's activity levels tend to diminish during the weekend [20,22-24]. Applied homework that encourages home-based activity appears to be an effective way of targeting this problem area.

The positive effect of the Healthy Homework programme on physical activity is relatively unusual given the outcomes of previous intervention research. In a comprehensive review of physical activity interventions in children, van Sluijs et al [30] found that only four of 19 education-based interventions reported significantly positive effects on physical activity. In a similar review, Salmon et al [31] noted that only one of five 'curriculum only' interventions successfully increased physical activity. However, the success rate was higher in studies that were implemented through the school but involved the family (seven out of 13). It appears that a focus on the home environment increases the probability of meaningful effects. Our promising results may have been due to the emphasis that was placed on increasing physical activity outside of school, including on the weekends.

Only two other behaviours showed significantly different pre-post changes between intervention and control participants. On weekends, vegetable consumption increased by 0.83 servings.day^-1 ^and unhealthy food consumption decreased by 0.56 servings.day^-1 ^as a result of programme participation. The increase in vegetable consumption is noteworthy as it is equivalent to approximately 28% of the daily vegetable recommendation of three servings a day. In addition, an increase of 0.83 servings.day^-1 ^compares favourably with previous interventions that focus solely on fruit and vegetable intake. Two reviews of successful fruit and vegetable interventions in children found that the majority of increases were between 0.2 and 0.6 daily servings [56,57]. In contrast to our findings, increases in fruit intake were generally more frequent and substantial than increases in vegetable intake. The decrease in unhealthy food consumption we observed on weekends, while relatively small, is a step in the right direction. Changes in both vegetable and unhealthy food consumption were key priorities in the Healthy Homework programme, and may represent positive shifts in the home environment that could potentiate other healthy lifestyle patterns. Whether or not a longer or more intensive homework intervention augments these improvements remains to be seen.

Non-significant effects in the remaining variables targeted in the intervention (screen time, sports participation, active transport to and from school, fruit consumption, and unhealthy drink consumption) suggest that the materials or approaches for these topics may have been insufficient. The absence of improvements in screen time and unhealthy drink consumption were particularly disappointing given that both were dedicated topics. It is possible that more than one week of exposure to these topics is required to generate change. Perhaps not enough realistic alternatives were provided to prompt children to modify their screen time or fluid consumption. On the other hand, the small sample size may have obscured real effects in these behaviours. Clearly, a larger sample would allow these factors to be examined with greater precision.

Another important discovery was that the intervention yielded benefits for boys and girls from a range of socioeconomic backgrounds. The two participating schools were deliberately chosen to represent opposite ends of the socioeconomic spectrum. The similarity of the intervention effects in both schools suggests that it is likely to be beneficial for other primary-level schools, regardless of the socioeconomic rating. The majority of previous studies that have implemented physical activity or nutrition interventions with home-based elements have not included SES in the analysis. Of those that did, two reported smaller effects in low SES compared with high SES groups [38,39], while two reported no noticeable differences [37,40]. Nonetheless, it is possible that in the latter studies (and the present one) the similar overall effects on physical activity and/or dietary patterns between SES groups were generated through different pathways. Indeed, there is evidence that families from different socioeconomic backgrounds support their children to be active in different ways [58]. A qualitative comparison of the preferences of homework activities and resources among children and parents from diverse socioeconomic regions could be beneficial in this regard.

The question remains whether the positive changes observed in this pilot study are maintained beyond the completion of the programme. A potential criticism of the programme was the use of wristbands as rewards to increase compliance: a viewpoint common among educators is that the desired behaviour will cease once the reward is removed. We contend that the rewards, in this instance, were used to engage children for the purpose of learning how to be active on their own. While this approach leans towards constructivism - the theory that individuals will generate their own knowledge and understanding from experience - it maintains enough structure that children with little or no understanding of the selected topics are guided towards discovery. The programme aims to create functional knowledge that is taken with the child beyond the completion of the programme, resulting in greater opportunities to be active and promoting lifelong healthy behaviour. Clearly, we cannot comment on the success of this ambition in the present pilot study; however, future studies should consider taking long-term follow-up measures to assess the sustainability of any positive outcomes. Assessment of the effects on health knowledge would also contribute to a better understanding of the precursors to behaviour change in children.

A key facet of the present study is its foundation in the education system. While the goals of the programme are clearly health-related, there are several advantages of operating within the education environment: (1) it is relatively cost-effective to introduce applied homework activities into an existing curriculum, (2) the vast majority of the population can be accessed (all children are required to attend school), and (3) the expertise of trained teachers can be utilised to effectively deliver health-related educational material and instruction. In this study, we developed a homework programme that contributed to all four strands of the Health and Physical Education achievement objectives stated in the New Zealand Curriculum [48]: (1) personal health and physical development, (2) movement concepts and motor skills, (3) relationships with other people, and (4) healthy communities and environments. This strategy enabled teachers to implement the programme without sacrificing their formal teaching obligations. Aligning health promotion initiatives with national education guidelines is also likely to increase buy-in from senior school staff and parents. Another important element of the study was its compulsory nature. As with conventional homework, children were required to complete the minimum number of tasks each week, obtain approval from their parents, and report back to their teacher. This approach was chosen to maximise the level of engagement in the programme, which may explain why significant effects on physical activity and diet were observed in a relatively small sample. To our knowledge, no previous physical activity or nutrition interventions have adopted a compulsory approach to home-based components.

The primary limitation of this study was the small sample size. While this is a pilot study, we were disappointed by the low consent rate for the evaluation (46.3%). Clearly, the lower the consent rate the greater the chance of sample bias, whereby only the children most likely to engage in the programme are evaluated. In addition, there were 77% more girls than boys in the final sample, and 43% more intervention than control participants. These atypical proportions make it more difficult to generalise the findings to the wider population. Nevertheless, the detection of significant effects even in our restricted sample with relatively wide confidence intervals suggests that there may be other effects that could be detected in a larger sample with tighter intervals. Another limitation was the necessity to randomise at the class level. It is probable that a certain amount of class contamination occurred, such that the behaviour of the control participants was affected by the experiences of the intervention participants as they progressed through the programme. Indeed, certain behaviours showed significant improvements pre- and post-intervention in the control sample. While the probability of class contamination does not negate the observed effects of the programme (true effects would be dampened rather than enhanced), it would be preferable for future studies to randomise at the school level. Also, we decided not to request the return of the children's booklets at the completion of the study (in case they were used in future), and consequently we had no record of homework compliance. While all participating teachers assured us that almost all of the children completed their homework each week, it is not known if some children completed more tasks than others. Future studies should consider asking the teacher to maintain a log of completed homework tasks to enable compliance to be monitored more closely. Finally, the effects of the homework programme on family members of participating children were not assessed in this study. Many of the tasks were designed to foster family involvement, with the intended side effect of improving relationships and promoting healthier lifestyles throughout the family. Further research is needed to elucidate these factors.

## Conclusions

Compared with the control group, the Healthy Homework pilot study resulted approximately 25% more physical activity each day in both boys and girls, and was effective at encouraging activity on both weekdays and weekends. Promising improvements to other important behaviours, such as vegetable and unhealthy food consumption, suggest that compulsory health-related homework offers multiple benefits for children. Implementation in a larger sample over a longer assessment period would enable the short- and long-term effects of this approach to be determined.

## Competing interests

The authors declare that they have no competing interests.

## Authors' contributions

SD conceived and designed the study, developed the intervention, contributed to the statistical analysis, and drafted the manuscript. JCM collected and entered the data and helped to draft the manuscript. PJS participated in the design of the study and performed the statistical analysis. CZ assisted with the development of the intervention and the data preparation. RS participated in the development of the intervention. GS helped design the study and develop the intervention. All authors reviewed and approved the final manuscript.
